# Precision of field triage in patients brought to a trauma centre after introducing trauma team activation guidelines

**DOI:** 10.1186/1757-7241-17-1

**Published:** 2009-01-09

**Authors:** Marius Rehn, Torsten Eken, Andreas Jorstad Krüger, Petter Andreas Steen, Nils Oddvar Skaga, Hans Morten Lossius

**Affiliations:** 1 Department of Research and Development, Norwegian Air Ambulance Foundation, Drobak, Norway; 2 Faculty of Medicine, Faculty Division Ulleval University Hospital, University of Oslo, Norway; 3 Department of Anaesthesiology, Aker University Hospital, Oslo, Norway; 4 Department of Anaesthesiology and Emergency Medicine, St. Olav University Hospital, Trondheim, Norway; 5 Prehospital division, Ulleval University Hospital, Oslo, Norway; 6 Department of Anaesthesiology, Division of Emergency Medicine, Ulleval University Hospital, Oslo, Norway

## Abstract

**Background:**

Field triage is important for regional trauma systems providing high sensitivity to avoid that severely injured are deprived access to trauma team resuscitation (undertriage), yet high specificity to avoid resource over-utilization (overtriage). Previous informal trauma team activation (TTA) at Ulleval University Hospital (UUH) caused imprecise triage. We have analyzed triage precision after introduction of TTA guidelines.

**Methods:**

Retrospective analysis of 7 years (2001–07) of prospectively collected trauma registry data for all patients with TTA or severe injury, defined as at least one of the following: Injury Severity Score (ISS) > 15, proximal penetrating injury, admitted ICU > 2 days, transferred intubated to another hospital within 2 days, dead from trauma within 30 days. Interhospital transfers to UUH and patients admitted by non-healthcare personnel were excluded. Overtriage is the fraction of TTA where patients are not severely injured (1-positive predictive value); undertriage is the fraction of severely injured admitted without TTA (1-sensitivity).

**Results:**

Of the 4 659 patients included in the study, 2 221 (48%) were severely injured. TTA occurred 4 440 times, only 2 002 of which for severely injured (overtriage 55%). Overall undertriage was 10%. Mechanism of injury was TTA criterion in 1 508 cases (34%), of which only 392 were severely injured (overtriage 74%). Paramedic-manned prehospital services provided 66% overtriage and 17% undertriage, anaesthetist-manned services 35% overtriage and 2% undertriage. Falls, high age and admittance by paramedics were significantly associated with undertriage. A Triage-Revised Trauma Score (RTS) < 12 in the emergency department reduced the risk for undertriage compared to RTS = 12 (normal value). Field RTS was documented by anaesthetists in 64% of the patients compared to 33% among paramedics.

Patients subject to undertriage had an ISS-adjusted Odds Ratio for 30-day mortality of 2.34 (95% CI 1.6–3.4, p < 0.001) compared to those correctly triaged to TTA.

**Conclusion:**

Triage precision had not improved after TTA guideline introduction. Anaesthetists perform precise trauma triage, whereas paramedics have potential for improvement. Skewed mission profiles makes comparison of differences in triage precision difficult, but criteria or the use of them may contribute. Massive undertriage among paramedics is of grave concern as patients exposed to undertriage had increased risk of dying.

## Background

Regional trauma care with designated trauma centres improve outcome for trauma patients [1-6]. Essential for these systems is field triage that identifies trauma victims with injury severity that justifies access to the documented benefits of trauma team resuscitation [7]. Some mistriage is unavoidable, given the evolutionary nature of symptoms following major trauma and that field triage is often performed in the early stages of care. Although physician input is known to increase triage precision [8,9], triage is often performed independently by paramedics with limited training in patient evaluation and structured triage decision-making. Imprecise field triage results in overtriage (trauma team activation (TTA) for the minimally injured patient) and undertriage (severely injured patient admitted without TTA). Priority has been to minimize undertriage, as it may result in adverse patient outcome due to denial of the potential benefits of immediate expert assessment and resuscitation provided by the trauma team. Although overtriage does not directly reduce patient safety, it results in overutilization of limited financial and human resources [10-12] and can cause reduced local emergency medical service (EMS) coverage [13]. As with any test, the cost of improved specificity will be reduced sensitivity. American College of Surgeons, Committee on Trauma (ACS-COT) [14] therefore describes 5% undertriage as acceptable and associated with an overtriage rate of 25% – 50%.

A wide range of trauma triage criteria have been proposed [2,5,15-17], but there is no consensus on the ultimate set of variables due to local variations in patient severity mix and trauma care organization. Still, many systems have partly adopted criteria proposed by ACS-COT [14], which focus on physiologic, anatomic and mechanistic parameters in addition to comorbidity. Although some of these criteria have been validated as predictors of severe injury [18-23] the majority remains without scientific evidence.

**Figure 1:**
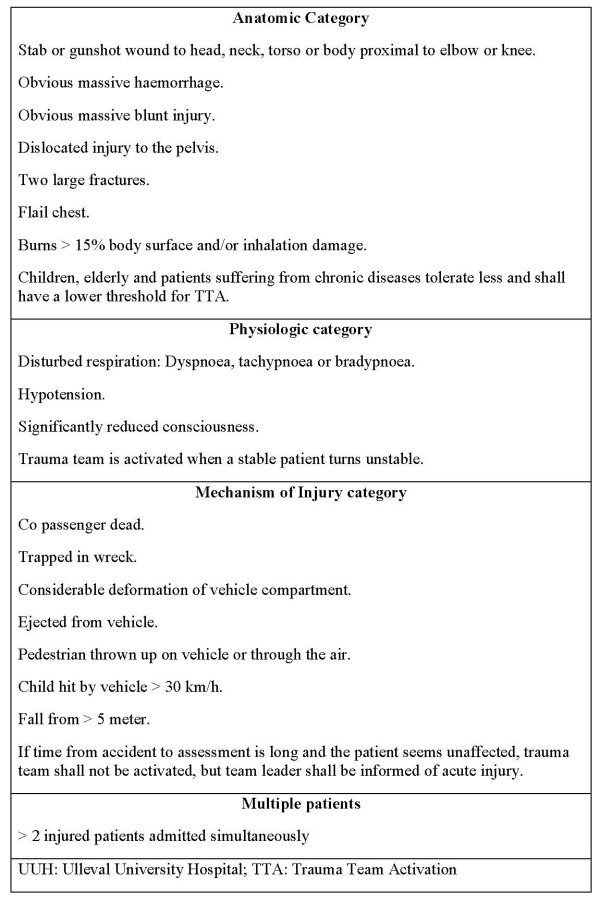
**Ulleval University Hospital trauma team activation (TTA) criteria**.

Ulleval University Hospital (UUH) is the largest trauma hospital in Norway and the trauma referral centre for half of the Norwegian population. Previously, UUH lacked a trauma triage protocol, and TTA was based on clinical judgment alone. In the year 2000, an analysis [9] found that the informal TTA system was imprecise with an undertriage of 11% and overtriage of 58% for primary admitted patients. Further, field triage was significantly more correct for patients admitted by anaesthetist-manned units than by paramedic-manned ambulances. This revealed an opportunity for improvement that catalysed the introduction of trauma triage guidelines (Fig. 1).

The continuous process of performance improvement as proposed by ACS-COT [14] refers to a cycle of monitoring, finding, fixing, and monitoring again. In order to close the loop, we wanted to describe triage precision among paramedics and anaesthetists after the introduction of the UUH TTA protocol. We also wanted to analyse how age, gender, category of prehospital care provider, vital signs, type of injury and triage criteria influenced triage precision.

## Methods

### Clinical background

UUH is the major trauma hospital for 550 000 and referral trauma hospital for 2.5 million people. The trauma team is one-tiered, with activation procedures partly based on guidelines published by ACS-COT (Fig. 1) [14]. Prehospital EMS units do not activate the trauma team directly, but report their findings to the ambulance dispatch centre. This information is immediately passed on to the nurse coordinator in the emergency department (ED) who activates the trauma team when at least one of four TTA criteria categories is fulfilled (Fig. 1). When in doubt, the nurse coordinator confers with the trauma team leader before TTA. Prehospital emergency care is provided by ordinary ambulance units staffed with paramedics and by anaesthetist-manned ground and air ambulances.

### Patients

**Figure 2:**
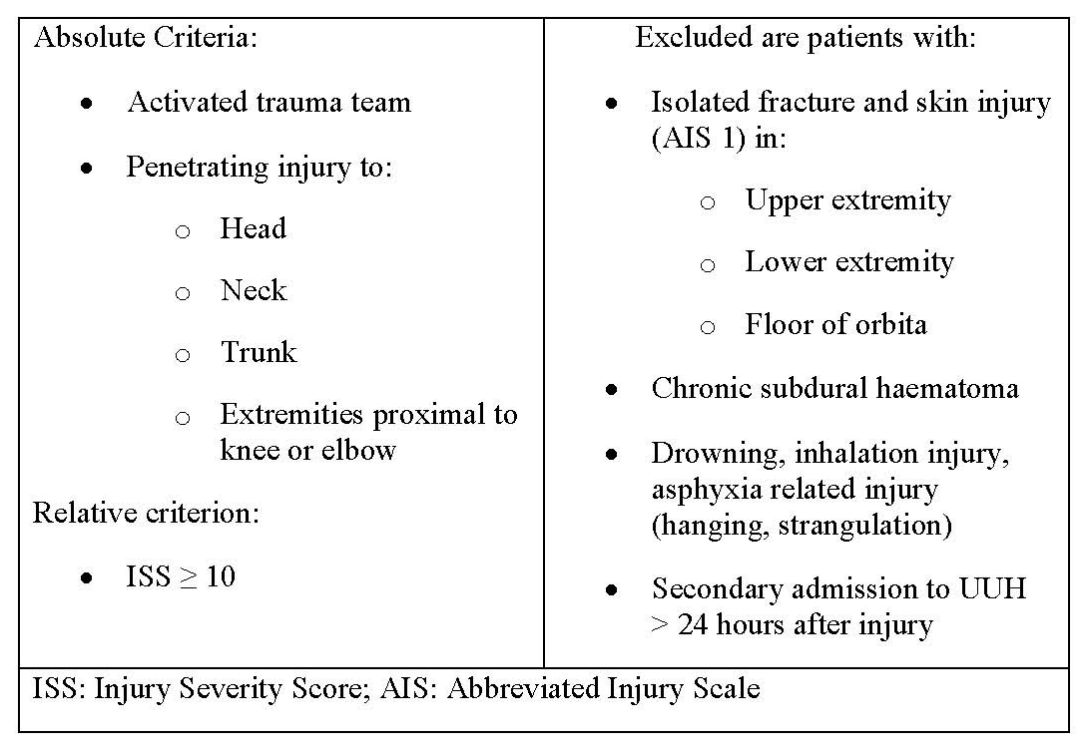
**Inclusion and exclusion criteria for the UUH trauma registry**.

We performed a retrospective analysis of prospectively collected data from the UUH trauma registry. The UUH trauma registrar utilizes a search engine to localize all patients with International Classification of Diseases (ICD) S- and T-codes from the hospital administrative system. This list is manually searched for relevant patients (see Fig. 2 for trauma registry inclusion and exclusion criteria). The study was exempted from the demand of informed consent due to anonymity of extracted data and the absence of any treatment study protocol, and the Regional Committee for Research Ethics and the Data Protection Official deemed approval as not necessary.

We included patients admitted to UUH during the period from 1^st ^of January 2001 to 31^st ^of December 2007, included in UUH trauma registry, and assigned one or more AIS codes (AIS 98; Abbreviated Injury Scale, 1990 Revision, Update 98) with an activated trauma team and/or severe injury. Patients were classified as severely injured if they fulfilled one of the following criteria: Injury Severity Score [24] (ISS) > 15; penetrating trauma to the head, neck, trunk, or extremities proximal to elbow or knee irrespective of ISS; need of intensive care for more than two days; transferred to another hospital intubated within two days; dead from trauma within 30 days. Interhospital transfers to UUH and patients transported by non-healthcare personnel were excluded, as they were not subject to UUH field triage guidelines.

30 days mortality was determined by information from the Norwegian Population Registry and hospital records [25]. Repatriated foreign citizens with inaccessible information on 30 days survival status were coded as survivors [26].

In patients who were prehospitally intubated and in general anaesthesia on hospital arrival, respiratory rate and Glasgow Coma Scale [27] (GCS) were scored according to values documented by the prehospital services immediately before intubation. In the absence of this information, we estimated the Triage – Revised Trauma Score (RTS) [28] category (0–4) of the variables respiratory rate and GCS score from the patient record, always utilizing the least pathological value when in doubt. In cases with complete lack of information, normal values were used as default [29].

### Statistical analysis

We assumed severely injured patients to potentially benefit from trauma team presence upon admission, and our evaluation of diagnostic precision of triage was based on this assumption. Various parameters can describe trauma triage precision. We defined ""Sensitivity" as the fraction of severely injured patients that were met by a trauma team (Table 1). "Undertriage" was defined as the contrary event, i.e. 1-sensitivity, interpreted as the probability of not being met by a trauma team despite being severely injured. To calculate specificity and thereby the classical definition of overtriage (1-specificity) [30], the number of patients with minor injuries admitted without TTA must be identified. As UUH each year receives a large number of primary admitted injured patients, the classical definition is of limited value. This sizeable and not easily definable group of patients is seldom considered for TTA, and would strongly bias the calculation of overtriage based on specificity. Optimal utilization of hospital resources requires a triage protocol that excludes minimally injured patients from TTA. Thus, "overtriage" was defined as the complement of the positive predictive value (1-PPV), where PPV represents the probability of a patient being severely injured when the trauma team is activated (Table 1) [9,31]. The null hypothesis that the TTA protocol did not improve triage precision was adopted. All data were analyzed using Statistical Package for the Social Sciences, v. 16.0 (SPSS, Inc., Chicago, IL). Data distributions are reported by medians and interquartile ranges (IQR). Non-parametric data were analysed with the Mann-Whitney test. For categorical data, the chi-square test was used and results are reported as odds ratios (OR) with 95% confidence interval (95% CI). We specifically wanted to study undertriage among severely injured patients, therefore undertriage was used as the dependent variable in the univariate and multivariate analyses. We used logistic regression to estimate the adjusted effects of each significant variable from the univariate analysis [31]. Variables were age, handled as a three level categorical variable (< 55, 55–70, > 70 years), whereas fall (yes, no), prehospital care provider (paramedic, anaesthetist), RTS (12, < 12) and gender were handled as dichotomous variables. ISS was handled as numerical value. Statistical significance was assumed for p < 0.05.

**Table 1:** Injury severity and trauma team activation (TTA)

	Severely injured	Not severely injured	Total
TTA	(a)	(b)	(a + b)
No TTA	(c)	(d)	(c + d)

Total	(a + c)	(b + d)	(n)

Sensitivity = a/(a + c); Specificity = d/(b + d)

Positive predictive value (PPV) = a/(a + b)

Undertriage = 1 - Sensitivity = c/(a + c); Overtriage = 1 - PPV = b/(a + b)

## Results

### Descriptive

During the study period, 4 885 patients were entered in the UUH trauma registry, of which 4 659 fulfilled our study inclusion criteria. Of the included patients, 4 208 (90%) had suffered blunt and 451 (10%) penetrating injuries as the dominant type of injury. Forty-two patients (1%) suffered both penetrating and blunt injury. Median age of included patients was 32 years (IQR 21 – 47), and median ISS was 9 (IQR 4 – 21).

### Clinical details of severely injured patients

Of the 4 659 patients, 2 221 (48%) fulfilled our criteria for being severely injured. A majority of these, 1 662 (75%), were men. Median ISS was 21 (IQR 14 – 29), with women having significantly higher ISS than men (median ISS 22 vs. 21, p = 0. 002). Median age was 36 years (IQR 23 – 53), with a significant difference in median age between the genders (women median 40 vs. men 34, p < 0.001).

### Precision in field triage

Among the 4 659 patients included, we recorded 4 440 (95%) activations of the trauma team. It was not activated for 219 of the 2 221 severely injured patients; an undertriage of 10%. The team was activated for minor injuries 2 438 times; an overtriage of 55%. Patients admitted by anaesthetist-manned units had 2% undertriage (among 1 059 severely injured patients, 25 received no TTA) and 35% overtriage (1 598 TTA where 564 were for minor injuries). Patients brought in by paramedics were subject to 17% undertriage (among 1 162 severely injured patients, 194 received no TTA) and 66% overtriage (2 842 TTA where 1 874 were for minor injuries) (Table 2). Among the 1 508 patients with TTA due to the mechanism of injury (MOI) criterion, 392 (26%) were severely injured (Table 3). The MOI criterion was used for 1 052 (37%) patients admitted by paramedics, compared to 456 (29%) of those admitted by anaesthetists (Table 4).

**Table 2:** Field triage precision by category of prehospital care before and after introduction of TTA protocol

	Without TTA protocol (1996)		With TTA protocol (2001 – 2007)	
	
	Overtriage	Undertriage	Overtriage	Undertriage
All patients	58%	11%	55%	10%
Anaesthetist admitted	44%	6%	35%	2%
Paramedic admitted	67%	17%	66%	17%

**Table 3:** Association and number of patients by category of prehospital care provider, TTA criteria, undertriage and correct triage

	Total	Severely injured	Dead within 30 days	Proximal penetrating injury	ICU > 2 days or transferred intubated	ISS > 15
Admission:						
Anaesthetist	1 623 (35%)	1 059 (65%)	185 (11%)	80 (5%)	756 (47%)	902 (56%)
Paramedic	3 036 (65%)	1 162 (38%)	173 (6%)	372 (12%)	476 (16%)	739 (24%)
	
Total	4 659 (100%)	2 221 (48%)	358 (8%)	452 (10%)	1 232 (26%)	1 641 (35%)
						
Patients with TTA	4 440 (95%)	2 002 (45%)	316 (7%)	426 (10%)	1 154 (26%)	1 467 (33%)
TTA criteria:						
Anatomic	1 192 (27%)	702 (59%)	107 (9%)	235 (20%)	361 (30%)	452 (38%)
Physiologic	76 (2%)	42 (55%)	9 (12%)	12 (16%)	20 (26%)	28 (37%)
MOI	1 508 (34%)	392 (26%)	33 (2%)	4 (0%)	245 (16%)	324 (22%)
Multiple patients	8 (0%)	3 (38%)	0 (0%)	1 (13%)	1 (13%)	2 (25%)
Several	760 (17%)	504 (66%)	127 (17%)	62 (8%)	351 (46%)	430 (57%)
Unknown	896 (20%)	359 (40%)	40 (5%)	112 (13%)	176 (20%)	231 (26%)
						
Undertriage	219	219 (100%)	42 (19%)	26 (12%)	78 (36%)	174 (80%)
Correct triage	2 002	2 002 (100%)	316 (16%)	426 (21%)	1 154 (58%)	1 467 (73%)

ICU: Intensive Care Unit; ISS: Injury Severity Score; MOI: Mechanism of Injury

**Table 4:** Usage and performance of TTA criteria by category of prehospital care provider

	Paramedic			Anaesthetist		
TTA criteria	Total	Correct triage	Overtriage	Total	Correct triage	Overtriage

Anatomic	717 (25%)	372 (52%)	345 (48%)	475 (30%)	330 (70%)	145 (30%)
Physiologic	65 (2%)	33 (51%)	32 (49%)	11 (0%)	9 (82%)	2 (18%)
MOI	1 052 (37%)	163 (15%)	889 (85%)	456 (29%)	229 (50%)	227 (50%)
Multiple patients	6 (0%)	2 (33%)	4 (67%)	2 (0%)	1 (50%)	1 (50%)
Several criteria	354 (13%)	182 (51%)	172 (49%)	406 (25%)	322 (79%)	84 (21%)
No documented criteria	648 (23%)	216 (33%)	432 (67%)	248 (16%)	143 (58%)	105 (42%)

Total	2 842	968 (34%)	1 874 (66%)	1 598	1 034 (65%)	564 (35%)

MOI: Mechanism of Injury

### Factors associated with undertriage

Among the 2 221 severely injured patients, age was significantly associated with undertriage, with an adjusted odds ratio (OR) of 2.19 for those between 55 – 70 years of age (CI 1.45 – 3.31; p < 0.001) compared to those younger than 55 years. For those older than 70 years, adjusted OR for being undertriaged was 5.41 (CI 3.60 – 8.13; p < 0.001).

Gender per se was also associated with undertriage, with an OR of 1.91 (CI 1.43 – 2.56; p < 0.001) for women compared to men. This difference lost its significance when we adjusted for age, giving an OR of 1.25 for women (CI 0.89 – 1.77; p = 0.202), as females were strongly represented among those over 55 years of age. Admittance by paramedics was also significantly associated with undertriage with an adjusted OR of 5.84 (CI 3.73 – 9.13; p < 0.001) compared to admittance by anaesthetists. Further, fall was associated with undertriage, with an adjusted OR of 4.89 (CI 3.51 – 6.83; p < 0.001). Finally, a Triage – RTS < 12 in the ED reduced the risk for undertriage with an adjusted OR of 0.42 (CI 0.30 – 0.60; p < 0.001) compared to RTS = 12 (normal value). Field RTS was documented by anaesthetists in 64% of the patients compared to 33% among paramedics (p < 0.001). Factors associated with undertriage are outlined in Table 5.

**Table 5:** Triage outcome split by factors associated with undertriage among 2221 severely injured patients. Unadjusted and adjusted (for gender, age, category of prehospital care, ED-RTS and fall), estimates of odds ratio for undertriage with 95% CI and p values

	Correct triage (n = 2 002)	Undertriage (n = 219)	OR (95% CI)	Adjusted OR (95% CI)
Gender:				
Men	1 525 (76%)	137 (63%)	1.00	1.00
Women	477 (24%)	82 (37%)	1.91 (1.43 – 2.56)*	1.25 (0.89 – 1.77)†

Age:				
<55 years	1 595 (80%)	99 (45%)	1.00	1.00
55–70 years	261 (13%)	46 (21%)	2.84 (1.96 – 4.13)*	2.19 (1.45 – 3,31)*
>70 years	146 (7%)	74 (34%)	8.17 (5.78 – 11.54)*	5.41 (3.60 – 8.13)*

Admitted by:				
Anaesthetist	1 034 (52%)	25 (11%)	1.00	1.00
Paramedic	968 (48%)	194 (89%)	8.29 (5.42 – 12.69)*	5.84 (3.73 – 9.13)*

ED-RTS:				
12	1 035 (52%)	156 (71%)	1.00	1.00
<12	967 (48%)	63 (29%)	0.43 (0.32 – 0.59)*	0.42 (0.30 – 0.60)*

Fall:				
No	1 632 (82%)	81 (37%)	1.00	1.00
Yes	370 (18%)	138 (63%)	7.52 (5.59 – 10.11)*	4.89 (3.51 – 6.83)*

CI: Confidence Interval; OR: Odds Ratio; *: p < 0.001; †: p = 0.202;

ED-RTS: Revised Trauma Score in the Emergency Department

### The consequence of undertriage

Patients subject to undertriage had significantly higher mortality risk compared to those correctly triaged, with an OR adjusted for ISS of 2.34 (CI 1.59 – 3.43; p < 0.001) (Table 6).

**Table 6:** 30 day mortality by category of triage. Unadjusted and adjusted for ISS

		Dead within 30 days				
	Total	Number of patients	OR (95% CI)	p-value	Adjusted OR (95% CI)	Adjusted p-value

Correct triage	2 002	316 (16%)	1.00		1.00	
Undertriage	219	42 (19%)	1.27 (0.89 – 1,81)	p = 0.23	2.34 (1.59 – 3.43)	P < 0.001

OR: Odds Ratio; CI: Confidence Intervals; ISS: Injury Severity Score

## Discussion

Patients brought to UUH by anaesthetists had a satisfactory triage precision, with an undertriage of 2% and overtriage of 35%, whereas patients brought in by paramedics were subject to unacceptable mistriage, with an undertriage of 17% and overtriage of 66% (Table 2).

Although patients admitted by paramedics were associated with less injury severity compared to those admitted by anaesthetists (median ISS 5 vs. 17, p < 0.001) due to overtriage, they were subject to a significantly higher risk for undertriage (Table 5). These results indicate that both patients and the trauma system could profit from integrating the highest level of medical competence accessible into the triage process. However, comparison of these patient groups must be made with caution, as skewed mission profiles might contribute to the observed differences.

The overall trauma triage system performance at UUH was outside the recommendations stated in the ACS-COT guidelines [14], with an undertriage of 10% and overtriage of 55% (Table 2). However, comparison of triage rates must be made with care, as different definitions of what constitutes a suitable patient for TTA – frequently referred to as a "severely injured" patient – are applied. Injury severity is a continuum and the cut off has traditionally been arbitrary. Nevertheless, the definition is fundamental, as it determines the threshold for inclusion to the care given by an activated trauma team, and provides the retrospective standard against which the triage guidelines will be tested. The US Major Trauma Outcome Study [32] found that ISS > 15 was related to a mortality risk of at least 10%, and despite some well-documented limitations [33,34], this cut off has been widely applied to define severe injury. We addressed these limitations by including proximal penetrating injury, need for ICU care and death from trauma within 30 days [25]. To achieve comparability with a previous analysis [9], the need for urgent ED procedure or operative intervention [35,36] (e.g. damage control laparotomy) was excluded from our definition, highlighting that consensus among researchers regarding a common definition of "severely injured" is needed. The current study is a retrospective review of trauma registry data and as such has several limitations. It is subject to retrospective bias and incomplete data collection, and it is restricted to variables already defined in the trauma registry. Some of the predefined data points (e.g. TTA criteria) lack detail and thus limit analysis precision. Further, the seven years delay between guideline introduction and the study of its efficacy may be considered too long.

Patients admitted by ordinary ambulances were more frequently triaged to TTA due to MOI (Table 4). MOI criteria were generally unable to predict severe injury regardless of personnel category involved in the triage process (Table 3). MOI was introduced as criterion after retrospective studies [37-39] revealed that some blunt trauma scenarios were associated with significant victim injury, which might remain occult throughout the prehospital period. Although it was recognized that this criterion would yield over-utilization of trauma centre resources, a certain amount of overtriage was deemed necessary to avoid preventable trauma deaths [14]. Car safety design and the utilization of safety restraints has markedly improved since many of these studies were published, and other papers now confirm the association between MOI as single criterion for TTA and overtriage [13,40-43].

Our results are consistent with prior studies that show that physiological and anatomical trauma triage criteria are predictive of the need for TTA [13,18,19,21,23] (Table 3). In general, anaesthetists put more emphasis on vital signs, as evidenced by prehospital RTS [28] being documented for 64% of the patients compared to 33% among paramedics (p < 0.001). Unsurprisingly triage – RTS < 12 in the ED reduced the risk for undertriage (Table 5). The presence of abnormal vital signs after involvement in trauma may suggest significant haemorrhage and the need for evaluation by the trauma team. However, the absence of abnormal vital signs or obvious anatomic injury does not rule out severe injury. We believe that "physiologic derangement" and "anatomic injury" categories should be mandatory criteria for full TTA at UUH, whereas MOI and "comorbidity" should be downgraded to only activate a trauma team consisting of fewer members. In an attempt to deal with the burden of overtriage generated by excessive use of the MOI criterion several trauma centers have introduced tiered triage systems, and published their positive experiences with them [11,16,44-46].

Patients subject to undertriage had significantly higher mortality risk compared to those correctly triaged, when adjusted for injury severity (Table 6). Phillips and co-workers [47] described falls as the main aetiology behind severe injury among elderly (hip fractures were excluded form the study), and that triage criteria according to ACS-COT recommendations failed to identify these trauma victims. We found both falls and increasing age to be significantly associated with undertriage, but there was no significant difference between genders when adjusted for age (Table 5). Problems in the initial evaluation of the traumatized geriatric patient may contribute to an increased risk of undertriage. Misleadingly "normal" initial vital signs despite severe injury due to medication and an inability to launch normal physiologic responses have been suggested as contributing factors [22]. Elderly trauma patients have particularly high mortality, even with fairly minor or moderately severe injuries. Undertriage in this group probably contributes to an even higher mortality. Demetriades et al. [22] have suggested that age over 70 years alone should be a criterion for TTA. In a later paper, Demetriades and coworkers [48] found that activated trauma team and early intensive monitoring, evaluation, and resuscitation of geriatric trauma patients improved survival.

The present study was conceived to highlight the supposed advantages of a trauma triage protocol, but increased precision could only be demonstrated among anaesthetists (Table 2). Although the introduced guidelines were based on fairly well documented material [18-22], triage precision among paramedics did not improve and therefore camouflaged any possible benefit on total system precision. Further, we found examples of breeched guidelines such as EMS providers activating the trauma team from the field instead of via the trauma coordinator. Such failure of guideline adherence may also contribute to this unexpected lack of increased triage precision. These results indicate that paramedics need further training in evaluating trauma victims. We also call for improved routines in communicating patient data from EMS units to the nurse coordinator in the ED, with vital signs, obvious anatomic injury, injury mechanism and comorbidity to be ordinal reported. Further, nurse coordinators would benefit from additional training in triage decision-making.

## Conclusion

Evaluating vital signs and anatomic injury require competence, and anaesthetists performed field triage with higher precision than paramedics, who displayed an unacceptably high mistriage rate. We therefore failed to reject the null hypothesis about any benefit brought about by introducing a trauma triage protocol. The discrepancy between personnel categories amplifies the need for a user-friendlier triage protocol and increased competence in trauma patient evaluation among paramedics. Although MOI with its low prediction accuracy was extensively used as TTA criterion, this alone could not explain all the imprecision. The "physiologic" and "anatomic" criteria performed well. Our findings should be an incitement to design a two-tiered trauma triage protocol, and thereafter change provider behaviour through a well-documented implementation strategy.

## Competing interests

The authors declare that they have no competing interests.

## Authors' contributions

MR and HML conceived the study. MR, TE, AJK, NOS and HML designed the study. MR and AJK performed the data analysis. NOS and TE designed and developed the UUH trauma registry. MR drafted the manuscript. All authors interpreted data and critically revised the manuscript. All authors have read and approved the final manuscript
